# *MPL* mutations and palpable splenomegaly are independent risk factors for fibrotic progression in essential thrombocythemia

**DOI:** 10.1038/bcj.2016.98

**Published:** 2016-10-21

**Authors:** M Haider, Y C Elala, N Gangat, C A Hanson, A Tefferi

**Affiliations:** 1 Division of Hematology, Department of Medicine, Mayo Clinic, Rochester, MN, USA; 2 Division of Hematopathology, Department of Laboratory Medicine and Pathology, Mayo Clinic, Rochester, MN, USA

Essential thrombocythemia (ET) belongs to the World Health Organization (WHO) tumor category of myeloproliferative neoplasms (MPN).^1^ ET is characterized by clonal thrombocytosis and its diagnosis requires absence of molecular or morphologic evidence of other myeloid malignancies including chronic myeloid leukemia, polycythemia vera, primary myelofibrosis (PMF), myelodysplastic syndromes or myelodysplastic syndromes/MPN overlap.^2^ Among MPN, ET has the best prognosis with a median survival of over 30 years in patients younger than age 60 years.^3^ Life-threatening complications in ET include thrombotic events, leukemic transformation and fibrotic progression.^4^

Diagnostic criteria for post-essential thrombocythemia (ET) myelofibrosis (MF) were developed by the International Working Group for MPN Research and Treatment and include historical documentation of a World Health Organization (WHO)-defined ET diagnosis, presence of grade greater than two bone marrow fibrosis and two or more additional features including anemia, leukoerythroblastosis, palpable splenomegaly, elevated serum lactate dehydrogenase level and constitutional symptoms.^5^ The incidence of fibrotic progression in ET ranges from 0.1 to 1% at 5 years, 0.8 to 5% at 10 years and 4 to 11% at 15 years and reported risk factors include advanced age, male sex, anemia, leukocytosis, bone marrow reticulin fibrosis, increased serum lactate dehydrogenase, absence of *JAK2*V617F mutation and use of anagrelide therapy.^6^ However, risk factor assessment for post-ET MF in the past has been confounded by the inadvertent inclusion of patients with prefibrotic PMF and the lack of information on driver mutational status; we addressed these issues in the current study.

After approval by the institutional review board of the Mayo Clinic, Rochester, MN, USA study patients were identified from our institutional database of MPN, based on availability of information on spleen size at time of diagnosis. ET diagnosis was according to the 2008 WHO criteria.^7, 8^ Clinical data collected were from the time of diagnosis. Leukemic or fibrotic disease progression was annotated by the treating physician and reviewed by the authors wherever possible and in line with criteria set by WHO and the International Working Group for MPN Research and Treatment.^8^ Previously published methods were used for *CALR, JAK2* and *MPL* mutation analyses.^3^ Differences in the distribution of continuous variables between categories were analyzed by either Mann–Whitney or Kruskal–Wallis test. Patient groups with nominal variables were compared by *χ*^2^-test. Kaplan–Meier survival analysis was considered from the date of diagnosis to date of death (uncensored) or last contact (censored). MF-free and leukemia-free survivals were determined from the time of diagnosis to the time the events occurrence after diagnosis. Cox proportional hazard regression model was used for multivariable analysis.

A total of 557 patients with median age of 59 years (61% females) met study eligibility criteria for WHO-defined ET. Laboratory characteristics at presentation and driver mutational status are outlined in Table 1; 66% of the patients harbored *JAK2*, 21% *CALR*, 3% *MPL* mutations and 11% were wild-type for all three mutations (that is, triple-negative). Thirty-five (6%) patients displayed palpable splenomegaly at time of diagnosis and anemia (adjusted for sex and mostly mild with hemoglobin levels of >11 g/dl) was present in 26% of patients. At a median follow-up time of 105 months, 170 (31%) deaths, 43 (8%) fibrotic progressions and 17 (3%) blast transformations were documented.

In univariate analysis, MF-free survival was significantly and negatively affected by male sex, *MPL* mutations, palpable splenomegaly and anemia and not by any other mutation, leukocyte count, platelet count, age or thrombosis history (Supplementary Table 1). Multivariable analysis for MF-free survival confirmed the adverse effect of *MPL* mutations (hazards ratio 6.7, 95% confidence interval 2.3–19.7; *P*<0.001), anemia (hazards ratio 3.3, 95% confidence interval 1.7–6.4; *P*<0.001) and palpable splenomegaly (hazards ratio 2.3, 95% confidence interval 1.02–5.3; *P*=0.04) (Supplementary Table 1). Accordingly, an hazards ratio-weighted scoring system with 1 point for splenomegaly, 2 points for anemia and 3 points for *MPL* mutation was developed and effectively stratified patients into low-, intermediate- and high-risk for transformation into post-ET MF (Figure 1). Supplementary Table 1 also shows univariate and multivariable analysis of the above described clinical and molecular parameters on overall and leukemia-free survival and the factors adversely affecting overall survival, on multivariable analysis, included advanced age, male sex, anemia, leukocytosis and thrombosis history and those affecting leukemia-free survival included anemia only (Supplementary Table 1). In other words, neither palpable splenomegaly nor *MPL* mutations were found to be detrimental to overall or leukemia-free survival.

The current study is novel in its observation regarding the association of palpable splenomegaly and the risk of post-ET MF in WHO-defined ET. The particular observation is consistent with the typical phenotypic profile of MF and suggests the possibility of occult/prefibrotic PMF in some ET patients with palpable splenomegaly at time of diagnosis.^2^ The same scenario might be the case underlying the association between anemia and post-ET MF. We have previously^3^ recognized the significant association between post-ET MF and *MPL* mutations in ET and this is now confirmed in the current study to be independent of both anemia and splenomegaly. The particular observation suggests that *MPL* mutations might be more a molecular marker for PMF than they are for ET and this is consistent with their higher prevalence in PMF and the fact that such driver mutations are seldom acquired during disease evolution. Interestingly and consistent with this observation, in a large international study, the presence of *JAK2* mutation (and thus the absence of patients with other driver mutations including those with *MPL* mutations) was associated with a lower risk of post-ET MF.^9^ From a practical standpoint, the possibility of prefibrotic PMF should always be considered in ET patients with *MPL* mutations, palpable splenomegaly or otherwise unexplained anemia and ET patients with one or more of these risk factors should be closely followed for the development of post-ET MF.

## Figures and Tables

**Figure 1:**
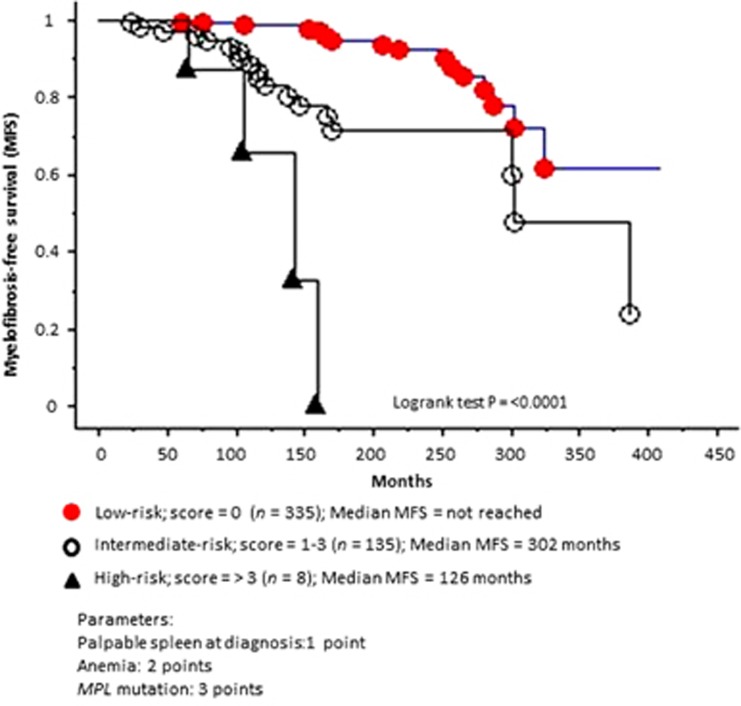
Myelofibrosis-free survival among 478 patients with essential thrombocythemia stratified into risk groups.

**Table 1:** Clinical and laboratory characteristics of 557 patients with essential thrombocythemia

*Patient characteristics*	*Total (*N=*557)*	*Palpable spleen at diagnosis (*n=*35)*	*No palpable spleen at diagnosis (*n=*522)*	P*-value*	*MPL mutated (*n=*12)*	*MPL unmutated (*n=*466)*	P*-value*
Median age (range), years	58.5 (14.3–96.4)	51.4 (18.3–85.1)	58.8 (14.3–96.4)	0.09	69.2 (58.6–87.0)	58.9 (14.338–96.422)	0.02
Age >60 years, *n* (%)	252 (45.2)	14 (40)	238 (45.6)	0.52	10 (83.3)	215 (46)	0.02
Female, *n* (%)	342 (61.4)	14 (40)	328 (62.8)	0.01	4 (33.3)	291 (62.4)	0.07
Median follow-up (range), months	105 (0.03–409.6)	118.7 (0.95–332.58)	103.5 (0.03–409.59)	0.29	95.376 (68.6–251.7)	112.9 (0.033–409.6)	0.92
Median platelet count (range), × 10^9^	862 (5–4000)	788 (266–3000)	864 (5–4000)	0.44	938.5 (654–2249)	879.5 (5–3401)	0.59
Median leukocyte count (range), × 10^9^	8.5 (1.3–53.4)	9.6 (3.4–21.4)	8.5 (1.3–53.4)	0.51	9.950 (3.6–32.6)	8.7 (1.9–53.4)	0.21
Median hemoglobin (range), g/dl	13.6 (6–17.9)	14 (8.4–16.7)	13.6 (6–17.9)	0.33	13.3 (9–15.8)	13.7 (6–17.9)	0.11
Anemia^a^, *n* (%)	143 (25.7)	12 (34.3)	131 (25)	0.23	7 (58.3)	110 (23.6)	0.01
Deaths, *n* (%)	170 (30.5)	14 (2.7)	156 (30)	0.21	8 (66.7)	154 (33)	0.03
Fibrotic transformation, *n* (%)	43 (7.7)	9 (1.7)	34 (6.5)	<0.001	4 (33.3)	35 (7.5)	0.01
Blastic transformation, *n* (%)	17 (3.1)	1 (2.9)	16 (3)	>0.99	1 (8.3)	15 (3.2)	0.34
Mutation: *CALR*/*JAK2*/*MPL*/triple-negative, %	20.9/65.5/2.6/11	15.6/68.8/3.1/12.5	21.3/65.3/2.5/10.9				
Palpable spleen at diagnosis, *n* (%)	35 (6.3)				1 (8.3)	31 (6.7)	0.57

a Anemia: hemoglobin level below the sex-adjusted lower limit of normal.
