# Supplementary material for: MPL mutations and palpable splenomegaly are independent risk factors for fibrotic progression in essential thrombocythemia
Source: Blood Cancer J. 2016 Oct 21;6(10):e487–. doi: 10.1038/bcj.2016.98 (PMC5098266; doi:10.1038/bcj.2016.98)
Supplement: Supplementary Table 1 [file bcj201698x1.docx]

| Fibrosis-free Survival: | | | | | | | | | | |  |
| --- | --- | --- | --- | --- | --- | --- | --- | --- | --- | --- | --- |
|  | **Univariate Analysis** | | | | | | | **Multivariable Analysis** | | |  |
| **Risk Factors** | | **Hazard Ratio** | | | **95% Confidence Interval** | **P** | **Hazard Ratio** | | **95% Confidence Interval** | **P** | |
|  | | |  | |  |  |  | |  |  | |
| **Age** | | | 1.01 | | 0.9-1.0 | 0.44 |  | |  |  | |
| **Female** | | | 0.54 | | 0.3-0.9 | **0.05** | 0.54 | | 0.3-1.1 | 0.07 | |
| **Mutational Status** | | |  | |  |  |  | |  |  | |
| *JAK2* | | | 0.98 | | 0.5-1.8 | 0.95 |  | |  |  | |
| *CALR* | | | 0.69 | | 0.3-1.5 | 0.33 |  | |  |  | |
| *MPL* | | | 8.25 | | 2.8-24.0 | **<0.001** | 6.68 | | 2.3-19.7 | **0.001** | |
| Triple Negative | | | 0.83 | | 0.3-2.2 | 0.71 |  | |  |  | |
| **Hemoglobin** | | | 0.79 | | 0.7-0.9 | **0.01** | 0.77 | | 0.6-0.9 | **0.004** | |
| **Anemia*** | | | 3.64 | | 1.9-6.7 | **<0.001** | 3.31 | | 1.7-6.4 | **<0.001** | |
| **Platelet count** | | | 1 | | 0.9-1.0 | 0.33 |  | |  |  | |
| **Leukocyte count** | | | 1.03 | | 1.0-1.08 | 0.22 |  | |  |  | |
| **Leukocyte count ≥ 11 x10^9** | | | 1.23 | | 0.7-2.3 | 0.52 |  | |  |  | |
| **Thrombosis at Diagnosis** | | | 0.86 | | 0.3-2.8 | 0.80 |  | |  |  | |
| **History of thrombosis** | | | 0.54 | | 0.2-1.73 | 0.30 |  | |  |  | |
| **Overall Survival:** | | |  | |  |  |  | |  |  | |
|  | | | **Univariate Analysis** | | | | **Multivariable Analysis** | | | | |
| **Risk Factors** | | | **Hazard Ratio** | | **95% Confidence Interval** | **P** | **Hazard Ratio** | | **95% Confidence Interval** | **P** | |
|  | | |  | |  |  |  | |  |  | |
| **Age** | | | 1.09 | | 1.1-1.1 | **<0.001** | 1.09 | | 1.2-1.1 | **<0.0001** | |
| **Female** | | | 0.65 | | 0.5-0.9 | **0.005** | 0.57 | | 0.4-0.8 | **0.0009** | |
| **Mutational Status** | | |  | |  |  |  | |  |  | |
| *JAK2* positive | | | 1.72 | | 1.3-2.4 | **<0.001** | 1.31 | | 0.9-2.0 | 0.205 | |
| *CALR* positive | | | 0.71 | | 0.5-1.0 | 0.07 |  | |  |  | |
| *MPL* positive | | | 2.37 | | 1.2-4.8 | **0.02** | 1.23 | | 0.5-2.7 | 0.609 | |
| Triple Negative | | | 0.44 | | 0.2-0.8 | **0.009** | 1.05 | | 0.5-2.1 | 0.900 | |
| **Hemoglobin** | | | 0.90 | | 0.8-0.98 | **0.02** |  | |  |  | |
| **Anemia*** | | | 1.71 | | 1.2-2.4 | **0.001** | 2.35 | | 1.6-3.4 | **<0.0001** | |
| **Platelet count** | | | 1 | | 1.0-1.0 | 0.86 |  | |  |  | |
| **Leukocyte count** | | | 1.05 | | 1.03-1.07 | **<0.001** |  | |  |  | |
| **Leukocyte count ≥ 11 x10^9** | | | 2.13 | | 1.6-2.9 | **<0.001** | 1.80 | | 1.3-2.5 | **0.0003** | |
| **Thrombosis at Diagnosis** | | | 1.21 | | 0.7-2.0 | 0.46 |  | |  |  | |
| **History of thrombosis** | | | 1.64 | | 1.1-2.4 | **0.01** | 1.50 | | 1.01-2.2 | **0.046** | |
| **Leukemia-free Survival:** | | | |  |  |  |  | |  |  | |
|  | | | **Univariate Analysis** | | | | **Multivariable Analysis** | | | | |
| **Risk Factors** | | | **Hazard Ratio** | | **95% Confidence Interval** | **P** | **Hazard Ratio** | | **95% Confidence Interval** | **P** | |
|  | | |  | |  |  |  | |  |  | |
| **Age** | | | 1.03 | | 1.0-1.2 | 0.07 |  | |  |  | |
| **Female** | | | 0.51 | | 0.2-1.3 | 0.17 |  | |  |  | |
| **Mutational Status** | | |  | |  |  |  | |  |  | |
| *JAK2* positive | | | 1.12 | | 0.4-2.9 | 0.82 |  | |  |  | |
| *CALR* positive | | | 1.16 | | 0.4-3.4 | 0.79 |  | |  |  | |
| *MPL* positive | | | 3.64 | | 0.5-28.2 | 0.22 |  | |  |  | |
| Triple Negative | | | 0.39 | | 0.1-3.0 | 0.36 |  | |  |  | |
| **Hemoglobin** | | | 0.90 | | 0.7-1.2 | 0.48 |  | |  |  | |
| **Anemia*** | | | 2.71 | | 1.02-7.2 | **0.05** | 2.81 | | 1.1-7.4 | **0.04** | |
| **Platelet count** | | | 1 | | 1.0-1.0 | 0.96 |  | |  |  | |
| **Leukocyte count** | | | 1.07 | | 1.0-1.1 | **0.01** |  | |  |  | |
| **Leukocyte count ≥ 11 x10^9** | | | 2.32 | | 0.9-6.0 | 0.08 | 2.42 | | 0.9-6.3 | 0.07 | |
| **Thrombosis at Diagnosis** | | | 0.73 | | 0.1-5.6 | 0.76 |  | |  |  | |
| History of thrombosis | | | 2.24 | | 0.7-6.9 | 0.16 |  | |  |  | |

*Anemia: Hemoglobin level below the sex-adjusted lower limit of normal.

**Supplementary table 1: Univariable and Multivariable analysis identifying risk factors impacting myelofibrosis- free survival, overall survival and leukemia-free survival in 557 patients with essential thrombocythemia**
